# Testing whether macroevolution follows microevolution: Are colour differences among swans (*Cygnus*) attributable to variation at the *MC1R *locus?

**DOI:** 10.1186/1471-2148-8-249

**Published:** 2008-09-12

**Authors:** Marie A Pointer, Nicholas I Mundy

**Affiliations:** 1 Department of Zoology, University of Cambridge, Downing St, Cambridge, CB2 3EJ, UK

## Abstract

**Background:**

The *MC1R *(melanocortin-1 receptor) locus underlies intraspecific variation in melanin-based dark plumage coloration in several unrelated birds with plumage polymorphisms. There is far less evidence for functional variants of *MC1R *being involved in interspecific variation, in which spurious genotype-phenotype associations arising through population history are a far greater problem than in intraspecific studies. We investigated the relationship between *MC1R *variation and plumage coloration in swans (*Cygnus*), which show extreme variation in melanic plumage phenotypes among species (white to black).

**Results:**

The two species with melanic plumage, *C. atratus *and *C. melanocoryphus *(black and black-necked swans respectively), both have amino acid changes at important functional sites in MC1R that are consistent with increased MC1R activity and melanism. Reconstruction of MC1R evolution over a newly generated independent molecular phylogeny of *Cygnus *and related genera shows that these putative melanizing mutations were independently derived in the two melanic lineages. However, interpretation is complicated by the fact that one of the outgroup genera, *Coscoroba*, also has a putative melanizing mutation at MC1R that has arisen independently but has nearly pure white plumage. Epistasis at other loci seems the most likely explanation for this discrepancy. Unexpectedly, the phylogeny shows that the genus *Cygnus *may not be monophyletic, with *C. melanocoryphus *placed as a sister group to true geese (*Anser*), but further data will be needed to confirm this.

**Conclusion:**

Our study highlights the difficulty of extrapolating from intraspecific studies to understand the genetic basis of interspecific adaptive phenotypic evolution, even with a gene whose structure-function relationships are as well understood as MC1R as confounding variation make clear genotype/phenotype associations difficult at the macroevolutionary scale. However, the identification of substitutions in the black and black-necked swan that are known to be associated with melanic phenotypes, suggests *Cygnus *may be another example where there appears to be convergent evolution at MC1R. This study therefore provides a novel example where previously described intraspecific genotype/phenotype associations occur at the macroevolutionary level.

## Background

A key question in evolutionary biology is whether the genetic basis of adaptations in macroevolution is the same as those of microevolution. This issue has been difficult to address since the different timescales involved lead to different methods of analysis, with population genetics being important for understanding microevolution and comparative phylogenetics being used for macroevolution. The growing understanding of the genetic basis of adaptive phenotypic traits, however, provides a new impetus to studies of this question.

Pigmentation is an excellent model for studying the genetic basis of phenotypic variation. In particular, melanization in vertebrates shows striking variability in pattern and colour that is frequently under strong natural or sexual selection, with the genetic control often well understood [1]. Many genes are involved in melanin production and deposition, however the melanocortin-1 receptor protein encoded at the *MC1R *locus plays a crucial role in controlling the type of melanin synthesized by melanocytes [2]. High activity of MC1R (either due to the presence of MSH agonist or high intrinsic MC1R activity) leads to synthesis of black/brown eumelanin, whereas low activity (due to an absence of MSH or presence of ASIP inhibitor) leads either to synthesis of reddish phaeomelanin, or an absence of melanin synthesis. Numerous studies have documented a strong association between colour variation within natural vertebrate populations and variations within the coding sequence of the *MC1R *locus [3-9]. One example is the lesser snow goose (*Anser c. caerulescens*), where the V85M substitution was found in one or both *MC1R *alleles in all melanic geese but was absent in white phase geese [6]. Interestingly, the same mutation was recently found to be associated with melanism in another bird, the red-footed booby (*Sula sula*) [10].

To date, almost all of the associations described between vertebrate coloration and *MC1R *have been intraspecific, and hence relating to microevolution. A previous study found no association between *MC1R *variation among species of Old World leaf warblers and the presence of small unmelanized patches [11], but it would have been surprising if such subtle plumage variation was affected by *MC1R *as polymorphisms known to associate with MC1R variation are more widespread across the pelage or plumage and not within small patches. A key question is whether *MC1R *variation plays a similar role in colour variation over longer evolutionary timescales as it is not yet clear whether similar or different molecular mechanisms underpin phenotypic variation at the macro- and microevolutionary scale. Methodologically, an absence of interbreeding means that the most direct means of demonstrating linkage/association between genotype and phenotype, i.e. in families or populations segregating for colour variation, is generally unavailable for interspecific studies. However, there is a good and growing understanding of MC1R structure-function relationships, which has arisen from *in vitro *studies of the biochemical function of MC1R [12] and studies of genotype-phenotype associations in domestic and wild species (for reviews see [13,1]. A crucial finding from these studies is that the same mutation leads to similar changes in MC1R function independent of other sequence variation in the protein. In addition, analyses of patterns of selection provide another route to identifying functionally important variation across phylogenies as demonstrated for MC1R and sexual dimorphism in galliformes [14].

**Figure 1:**
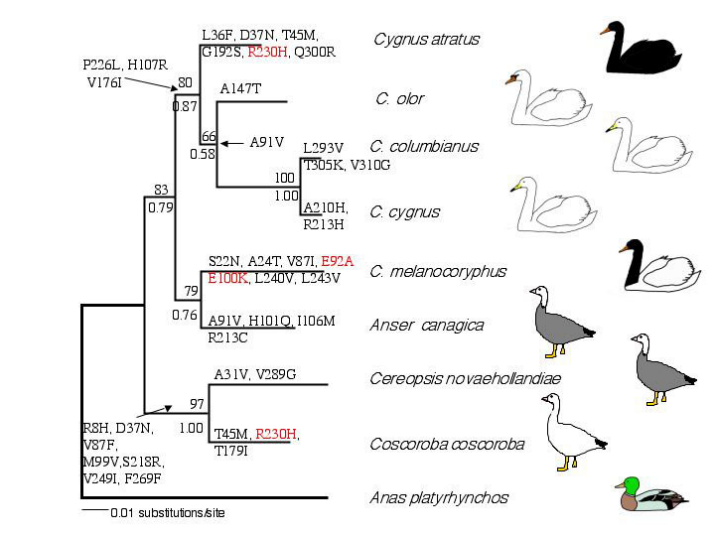
**Reconstruction of *MC1R *evolution over an independent molecular phylogeny of swans and geese**. A maximum likelihood tree of swans and geese using *CytB *and *LDH B *data. Amino acid substitutions at important functional sites in *MC1R *are shown in red, all other substitutions are shown in black. Numbers above branches are bootstrap support values from 100 replicates. Numbers below branches are the posterior support probabilities from Bayesian analysis. *Anas platyrhynchos *sequences were obtained from GenBank; Accession numbers EU009397 (*CytB*) and X68810 (*LDH B*).

The swans (*Cygnus*) provide a simple system of colour variation for investigating whether *MC1R *has a role in interspecific colour differences, and are in the same family as the lesser snow goose which shows intraspecific association between MC1R genotype and plumage melanism. All swan species are sexually monomorphic. The four species in the Northern hemisphere, *Cygnus olor *(mute swan), *C. cygnus *(whooper swan), *C. buccinator *(trumpeter swan) and *C. columbianus *(tundra swan) display pure white plumage. In the Southern hemisphere, *C. atratus *(black swan) has completely black plumage and *C. melanocoryphus *(black-necked swan) has white plumage on the torso and wings with black feathers on the head and neck (Figure 1).

In this study we address whether *MC1R *is associated with melanin-based plumage coloration in *Cygnus*. We determine whether variation at important functional sites in *MC1R *is associated with colour variation, and whether these variants are evolutionarily primitive or derived. In order to do this, we reconstruct MC1R evolution over a newly generated, independent molecular phylogeny of the genus *Cygnus*, since phylogenetic relationships both within the genus and among *Cygnus *and other genera of geese and swans (*Anser*, *Cereopsis*, *Coscoroba*) are poorly understood [15]

## Results

### Phylogenetic reconstruction of swans and geese using *CytB *and *LDH B *data

In total an 812 bp alignment (502 bp *Cytochrome B*, 310 bp *Lactate dehydrogenase B*) of all taxa was obtained. In MODELTEST, the TrN + I + G model [16] provided the best fit to the data. The maximum likelihood reconstruction obtained with this model in PAUP is shown in Figure 1. The identical topology was recovered in Bayesian analyses, and bootstrap support percentages and Bayesian support strengths are commensurate. Surprisingly, the genus *Cygnus *is not recovered as a monophyletic clade: four of the *Cygnus *species form a well-supported monophyletic group but *C. melanocoryphus *is placed as a sister group to *Anser *with moderate support. Elsewhere on the phylogeny, the coscoroba swan and the Cape Barren goose are strongly supported as sister taxa, which has previously been suggested based on the presence of a CR1 retrotransposon in *LDH B *[17].

### *MC1R *variation and reconstruction in *Cygnus*

The full 945 bp coding sequence of *MC1R *was obtained for all species studied. There was substantial variation in the open reading frame among different species of *Cygnus*, with 19 synonymous and 33 non-synonymous variable positions found. All five species had unique predicted protein sequences. Sites of functional importance were identified from previous studies based on linkage/association between MC1R variants and coat colour, and *in vitro *studies of MC1R function, including site-directed mutagenesis. Three putatively important mutations (at sites 92, 100 and 230) were identified among *Cygnus *species: one of these (230H) occurs in *C. atratus*, two (92A, 100K) occur in *C. melanocoryphus*, and none occur in any of the white species. Reconstruction of *MC1R *evolution over the molecular phylogeny in PAML shows that these mutations were derived in terminal lineages (Figure 1).

All five individuals of *C. atratus *sampled have an R230H substitution. The same substitution is associated with melanism in arctic skuas (*Stercorarius parasiticus*) [6] and a histidine at this position is also associated with melanism in rock pocket mice (*Chaetodipus intermedius*) [18].

*Cygnus melanocoryphus *has two putative functional mutations that are both associated with a change of charge, an E92A mutation and an E100K mutation. A charge-changing mutation at the position homologous to 92 (E92K) is associated with melanism in mice, chicken, quail and bananaquit (*Coereba flaveola*)[19-21,8] and *in vitro *studies in mouse and chicken have shown that the mutation causes constitutive activation of MC1R [19,22]. The E100K mutation occurs at a strongly conserved site that when deleted is associated with melanism in jaguar and jaguarundi [4].

### *MC1R *variation in other species, and tests of selection

Among other taxa studied, only *Coscoroba *had a mutation at an important functional site in MC1R. *Coscoroba *shares a derived R230H substitution with *C. atratus*, and maximum likelihood reconstruction over the phylogeny using PAML predicts an R residue at position 230 in the inferred last common ancestor between *Coscoroba *and *Cygnus *(posterior probability = 0.997) strongly suggesting that the R230H substitution occurred independently in the two lineages.

Lineage-specific analyses in PAML showed that the melanic *C. atratus *and *C. melanocoryphus *had a higher estimated dN/dS ratio (0.21) than other *Cygnus *lineages (0.12), but this was not significant. Site-specific analyses over the whole phylogeny did not provide any evidence for a category of sites under positive selection (not shown).

## Discussion

### *MC1R *variation and evolution of plumage coloration in swans and geese

Within the genus *Cygnus*, it is striking that the only variation at known sites of functional importance in MC1R was found in the two melanic species, *C. atratus *and *C. melanocoryphus*. These species both had independent derived mutations (R230H in *C. atratus*, E92A and E100K in *C. melanocoryphus*) consistent with an increase in melanism. Taken at face value, this suggests that MC1R is of functional importance for melanism among species of *Cygnus*, and also suggests that the ancestral swan may have been white.

However, the interpretation is clearly complicated by the finding of the R230H mutation in *Coscoroba*, which has close to pure white plumage. Either the R230H mutation has no influence on plumage coloration or it has a differential effect on phenotype in *C. atratus *and *Coscoroba*. The association of a 230H with melanism in two distantly related species (Arctic skua and rock pocket mouse) strongly suggests that this mutation does influence MC1R function. In addition, a growing body of evidence from *in vitro *and association studies shows that mutations in the same positions of MC1R generally have similar effects on MC1R function in very different lineages, i.e. independently of other variation at the locus. For example, an E92K mutation has been shown to cause constitutive activation (high activity in the absence of MSH) in natural MC1R variants of mouse, chicken and red-ruffed lemur (*Varecia rubra*) [19,22,12] as well as human MC1R subjected to *in vitro *mutagenesis [23] Another example is the R65C mutation, which leads to low MC1R activity in beach mice (*Peromyscus polionotus*) and mammoth [24,25]. Taken together, the presence of histidine at position 230 is therefore likely to affect MC1R activity in *C. atratus *and *Coscoroba*.

We therefore suggest that the most likely explanation for our results is that MC1R activity is increased in both *C. atratus *and *Coscoroba*, but whereas in *C. atratus *this leads to the expected melanism, in *Coscoroba *other genetic variation, e.g. at one or more loci downstream of MC1R, influences the phenotype. A precedent for the situation in *Coscoroba *is found in the red-ruffed lemur (*Varecia rubra*). This primate has a large amount of phaeomelanin in the coat but also an E94K mutation (homologous to position 92 in the swans), which is generally associated with large amounts of eumelanin [26]. Recent *in vitro *studies have shown that this mutation leads to the expected constitutive activation in MC1R, but that in this case the MC1R is still responsive to the inhibitor ASIP, so regulation of ASIP is almost certainly involved in the phenotype [12].

### Reconstruction of *Cygnus *phylogeny

This is the first time to our knowledge that the relationships within the *Cygnus *genus have been investigated at the molecular level. Surprisingly, our results do not support the monophyly of *Cygnus *but suggest that *C. melanocoryphus *is more closely related to the emperor goose than to the remaining *Cygnus *species. These results conflict with a consensus parsimony tree obtained from 165 morphological traits in modern and fossil Anserinae [27] in which *Cygnus *was monophyletic, with *C. melanocoryphus *a sister taxon to *C. atratus*. Interestingly, *C. melanocoryphus *was first classified, not as *Cygnus*, but as the sole member of the genus *Sthenelides *[28]. However, the results are based on two loci, and resolution is not strong throughout the phylogeny, and further molecular data and denser sampling of other true geese (*Anser *and *Branta*) will be required to increase the resolution of the *Cygnus *phylogeny. However, it is important to note that the placement of *C. melanocoryphus *does not affect the conclusions of the MC1R analysis.

Elsewhere on the phylogeny, the coscoroba swan is removed from the *Cygnus *and emperor goose clade, and instead forms a strongly supported clade with the Cape Barren goose, a grouping that has received previous support in molecular datasets (CR1 transposon in *LDH B *[17], mitochondrial srRNA [29]). In contrast, in a recent analysis of the Anseriformes based on mtDNA control region sequences, a clade containing *Cygnus*, *Coscoroba *and *Cereopsis *to the exclusion of *Anser *was recovered, although statistical support for this grouping was rather weak [15].

## Conclusion

We have likely uncovered an example where macroevolutionary change in plumage colour among species of *Cygnus *involves variation at the same locus that is involved in microevolution of melanism within a species of *Anser*, the snow goose *A. caerulescens*. However, the finding of a predicted melanizing mutation in MC1R in *Coscoroba *highlights the problems of providing a clear demonstration of functional involvement of a locus in a phenotype in cases where crosses/transgenics are not possible. A particular difficulty is that even though there is good information on the structure-function relationships of MC1R, and growing evidence that point mutations have effects on function that are largely independent of other sequence variation, the biochemical properties of MC1R do not necessarily predict the overall colour phenotype with epistatic interactions with other pigmentation loci also being key.

## Methods

### Sampling and DNA extraction

Samples comprised: muscle stored in ethanol from *Cygnus olor *(mute swan), *C. cygnus *(whooper swan), *C. columbianus *(tundra swan), *Coscoroba coscoroba *(coscoroba swan), *Anser canagica *(emperor goose) and *Cereopsis novaehollandiae *(cape barren goose) (National Museum of Scotland, Edinburgh); muscle stored in RNAlater (Ambion) from *C. melanocoryphus *(Natural History Museum, London); genomic DNA from five *C. atratus *individuals (Raoul Mulder, University of Melbourne). Genomic DNA was extracted using the QIAamp DNA Mini Kit (Qiagen) following the manufacturer's protocol.

### Genomic DNA walking and sequencing of *MC1R*

The 3' UTR of *MC1R *from *C. atratus *was obtained using the BD GenomeWalker™ Universal Kit (BD Biosciences Clontech) according to manufacturer's instructions. Briefly, 2.5 μg of genomic DNA was digested with each of the four restriction enzymes; DraI, EcoRV, PvuII and StuI and ligated to the BD genomewalker adaptor. Primary PCRs were performed using the adaptor AP primer (kit) and 3' Swan O (5'-CCAACCCCTTCTGCAACTGCTTCTTCGG-3') obtained from preliminary partial *MC1R *sequences of *C. atratus*. PCR reactions were performed in 50 μl, with 2.5 U polymerase (Extensor Hi-Fidelity PCR Master Mix: Abgene), 0.4 μM of each primer and 1 μl adapted DNA. The cycling conditions were: 7 × 94°C 25 s, 72°C 3 min.; 32 × 94°C 25 s, 67°C 3 min.; 67°C 7 min. Secondary PCRs were performed using primers AP2 (kit) and 3'swan inner (5'-CAACCTCTTCCTCATCCTCATCATCTGC-3'). A 1.3 kb segment of *MC1R *including the entire coding region was amplified using a forward primer designed to the 5' UTR of the chicken *MC1R *gene (Chick 5' utr; 5'-GAGAAAGGGCCCTTTCTTC-3') and a reverse primer designed to the isolated 3' UTR of the *C. atratus MC1R *gene (Swan 3' utr; 5'-TAGCCTTTATTCGGTACCG-3') in a PCR containing 2.5U taq (Extensor Hi-Fidelity PCR Master Mix, Abgene), 2.25 mM MgCl_2_, 350 μM dNTPs, 0.2 μM of each primer and 50 ng DNA with cycling conditions of 94°C 3 min, 40 × 94°C 30 s, 62°C 45 s, 70°C 45 s; 70°C 5 min. For some samples, a new 5' UTR primer (Swan 5' utr; 5'-GCCCATGTCCTCTTGACC-3') was designed that bound downstream of the chicken 5' primer. PCR products were sequenced on both strands with BigDye v. 3.1 (Applied Biosystems) using PCR primers and the internal primers MSHR73 (5'-GGCGTAGAAGATGGTGATGTAGC-3') and MSHR74 (5'-GTGGACCGCTACATCACCAT-3').

### Cytochrome B (*CytB*) and Lactate Dehydrogenase B (*LDH B*) sequencing

PCR primers Cyt B+ (5'-TGCCGACCTGCAAGTAGC-3'), Cyt B- (5'-GCGATTGAGGCGAGTTGG-3'), LdhF (5'-GGAAGACAAACTAAAAGGAGAAATGATGGA-3') and LdhR (5'-TTCCTCTGAAGCAGGTTGAGACGACTCTC-3') were designed to conserved regions identified in avian *CytB *and *LDH B *sequences available on GenBank. PCRs were performed with standard Taq and reagent concentrations. For Cytb, PCR conditions were: 94°C 3 min.; 35 × 94°C 30 s, 69°C 30 s, 70°C 45 s; 70°C 5 min. A product of approximately 1 kb was sequenced with the PCR primers and the two internal primers CytB 200-(5'-AGGCGAGTGAGGTGTCTG-3') and CytB 700-(5'-ATTTTGTCGCAGTCTGAYACG-3'). Sequences of 502 bp were obtained for all samples. For *LDH B*, PCR cycling conditions were: 94°C 2 min.; 35 × 94°C 1 min, 55°C 1 min, 72°C 2 min.; 72°C 5 min. The 600 bp products were sequenced with the PCR primers and the internal primers Ldh 390R (5'-AACTGCCACCCAAAAYAG-3'), Ldh 450F (5'-TAGTACTTTAATATAGAAG-3') and Ldh 750R (5'-TGACGAACACCTGCAGTTAC-3') providing sequences of 310 bp for all samples.

(*CytB*, *LDH B *and *MC1R *sequences have been deposited in GenBank, Accession numbers FJ170040–FJ170063).

### Phylogenetic analysis

Sequences of *CytB *and *LDH B *were concatenated and aligned with CLUSTAL-W [30] using MegAlign software (DNAstar). Positions containing gaps were removed before further analysis. Using MODELTEST [31], the model of sequence evolution with the highest maximum likelihood score as shown by the Akaike Information Criterion method was obtained and then implemented in maximum likelihood analyses in PAUP (v. 3.1, [32]). Branch support was examined by bootstrap resampling [33] with 100 replicates. Bayesian inference of phylogeny was performed in MrBayes (v. 3.1.2, [34]. The data were partitioned into CytB and LDH B and a GTR + I + Γ model of evolution applied to each partition, and 500,000 generations run, with sampling every 100 generations. Reconstruction of MC1R amino acid substitutions over the phylogeny obtained was performed in PAML (v.3.13, [35]).

### Tests for Selection

For the *MC1R *dataset, dN/dS ratios were estimated in PAML using a codon-based substitution model. Two separate analyses were performed: 1). Lineage-specific models where the dN/dS was estimated for lineages with and without melanism, i.e. *C. atratus *and *C. melanocoryphus *to the rest of the species analysed. 2). Site specific models in which different codons are grouped into dN/dS categories across all lineages. Likelihood ratio tests were performed on nested sets of analyses.

## Authors' contributions

MAP carried out all lab work and performed MODELTEST, PAUP and PAML analyses. NIM conceived of the study and performed the MrBayes analysis. Both authors were involved in the experimental design and wrote and approved the final manuscript.
